# Staphylococcal Cassette Chromosome *mec* in MRSA, Taiwan

**DOI:** 10.3201/eid1303.060247

**Published:** 2007-03

**Authors:** Jann-Tay Wang, Chi-Tai Fang, Yee-Chun Chen, Chia-Ling Wu, Mei-Ling Chen, Shan-Chwen Chang

**Affiliations:** * National Taiwan University Hospital, Taipei, Taiwan, Republic of China

**Keywords:** Methicillin-resistant *Staphylococcus aureus*, multilocus sequence typing, SCC*mec*, dispatch

## Abstract

To determine the predominant staphylococcal cassette chromosome (SCC) *mec* element in methicillin-resistant *Staphylococcus aureus*, we typed 190 isolates from a hospital in Taiwan. We found a shift from type IV to type III SCC*mec* element during 1992–2003, perhaps caused by selective pressure from indiscriminate use of antimicrobial drugs.

The high prevalence of methicillin-resistant *Staphylococcus aureus* (MRSA), which accounts for as much as 80% of all *S*. *aureus* isolates causing nosocomial infections in Taiwanese hospitals since 1998, has greatly affected infection control and medical treatment in Taiwan (*1*). At National Taiwan University Hospital (NTUH), a 2,200-bed major teaching hospital in northern Taiwan, MRSA has become a common nosocomial pathogen since the early 1990s. The annual number and incidence of nosocomial MRSA infections, as well as the number of available nonduplicate isolates for the past 12 years at NTUH, are shown in the Figure.

**Figure:**
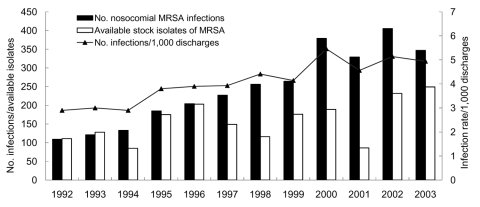
Number and cumulative incidence of nosocomial methicillin-resistant *Staphylococcus* *aureus* (MRSA) infections per 1,000 discharges and number of available nonduplicate MRSA isolates at National Taiwan University Hospital, 1992–2003.

In a previous study, we used pulsed-field gel electrophoresis typing of 140 randomly selected nosocomial MRSA isolates and samples from our collection of nosocomial isolates obtained from 1992 to 1996 to identify 3 major pulsotypes (A, B, and C) (*2*). Pulsotype A was predominant among all MRSA isolates in 1992 (accounting for 50%) and 1993 (52%), pulsotype B was predominant in 1994 (59%) and 1995 (49%), and pulsotype C was predominant in 1996 (83%) (*2*). Pulsotype C remained the predominant clone until 2003 (J.-T. Wang et al., unpub. data).

*S*. *aureus* acquires methicillin resistance through a mobile staphylococcal cassette chromosome (SCC) that contains the *mecA* gene complex (SCC*mec*) (*3*). Until now, 5 major types of SCC*mec* element have been characterized and studied (*3*–*5*). However, longitudinal studies of SCC*mec* elements in nosocomial MRSA isolates in a hospital have seldom been reported (*6*). We analyzed SCC*mec* elements in predominant nosocomial MRSA clones at NTUH from 1992 through 2003. Because the predominant MRSA clone at NTUH from 1996 through 2003 was pulsotype C, MRSA isolates obtained from 1997 through 2002 were not studied.

## The Study

We analyzed all 140 MRSA isolates we obtained during a previous study (*2*) and 50 other isolates selected from our collection of nosocomial MRSA isolates obtained in 2003. Characteristics of these 190 isolates are shown in Table 1.

**Table 1:** Pulsotypes, characteristics, and sources of 190 methicillin-resistant *Staphylococcus aureus* (MRSA) isolates, Taiwan, 1992–2003

Year, clinical syndrome (no. isolates)*	Pulsotype (no. isolates)	Source specimen (no. isolates)†	Site of isolation (no. isolates)‡
1992			
NI (9)	A (4), B (3), F (1)	Bl (3), Ur (2), Sp (2), Pu (2)	ICU (6), ward (3)
NC (8)	A (3), B (4), F (1)	Sp (4), Wo (4)	ICU (3), ward (5)
IEOH (5)	A (4), B (1)	Sp (4), Pu (1)	ICU (3), ward (2)
1993			
NI (10)	A (1), B (8), F(1)	Bl (4), Ur (1), Sp (2), Pu (3)	ICU (8), ward (2)
NC (14)	A (11), B (3)	Sp (5), Wo (5), Ns (3), Ct (1)	ICU (9), ward (5)
IEOH (1)	A (1)	Sp (1)	Ward (1)
1994			
NI (9)	A (4), B (4), D (1)	Bl (7), Sp (1), Pu (1)	ICU (4), ward (5)
NC (8)	A (1), B (6), C (1)	Sp (4), Wo (3), Ns (1)	ICU (5), ward (3)
1995			
NI (9)	B (3), C (5), E (1)	Bl (2), Sp (3), Pu (4)	ICU (6), ward (3)
NC (25)	A (1), B (13), C (9), D (2)	Sp (14), Wo (5), Ns (6)	ICU (19), ward (6)
IEOH (1)	B (1)	Sp (1)	Ward (1)
1996			
NI (23)	A (1), B (1), C (20), D (1)	Bl (15), Sp (3), Pu (5)	ICU (16), ward (7)
NC (11)	A (1), C (10)	Sp (7), Wo (2), Ns (1), St (1)	ICU (11)
IEOH (7)	A (1), B (2), C (4)	Bl (2), Sp (5)	ICU (5), ward (2)
2003			
NI (24)	C (10), D (2), K (5), L (2), M (3), other (2)	Bl (16), Sp (4), Pu (4)	ICU (10), ward (14)
NC (26)	A (1), C (10), K (6),L (3), M (5), other (1)	Sp (14), Wo (12)	ICU (12), ward (14)

*NI, nosocomial infection acquired at National Taiwan University Hospital (NTUH); NC, nosocomial colonization at NTUH; IEOH, MRSA infection detected at NTUH within 48 h after transfer from another hospital. †Bl, blood; Ur, urine; Sp, sputum; Pu, pus; Wo, wound; Ns, nostril; Ct, catheter tip; St, stool. ‡ICU, intensive care unit.

Pulsed-field gel electrophoresis patterns were interpreted according to procedures previously reported (*7**,**8*). Thirty-four isolates belonged to pulsotype A, 49 to pulsotype B, 69 to pulsotype C, 6 to pulsotype D, 2 to pulsotype E, 3 to pulsotype F, 11 to pulsotype K, 5 to pulsotype L, 8 to pulsotype M, and 3 (all isolated in 2003) to 3 minor pulsotypes. All isolates were tested by SCC*mec* element typing and multilocus sequence typing (MLST) (*9*) and were analyzed for the Panton-Valentine leukocidin (PVL) gene (*10*) and drug susceptibility to erythromycin, clindamycin, gentamicin, amikacin, ciprofloxacin, levofloxacin, tetracycline, trimethoprim-sulfamethoxazole, rifampin, and vancomycin by using the disk diffusion method (*11*). SCC*mec* element typing was determined by previously described PCR methods (*3*–*5*).

Results of these analyses are shown in Table 2. MRSA isolates of the same pulsotype have the same MLST pattern and SCC*mec* types. Isolates with pulsotype A are sequence type 254 (ST254); those with pulsotype B are ST241; those with pulsotypes C, K, and L are ST239; and those with pulsotypes D, E, F, and M are ST59, ST 254, ST30, and ST5, respectively. All MRSA isolates with pulsotypes A, D, E, and F have the type IV SCC*mec* element. However, only isolates with pulsotypes D and F, as well as 2 isolates from 2003 with 2 minor pulsotypes, have the PVL gene. Isolates with pulsotypes B and C have the type III SCC*mec* element, and isolates with pulsotype M have the type II SCC*mec* element.

**Table 2:** Characteristics of 190 methicillin-resistant *Staphylococcus aureus* isolates, Taiwan, 1992–2003*

P (no. isolates)	SCC *mec* type	MLST	PVL		Year of isolation (no. isolates)	Drug susceptibility rate, %†										
						OX	EM	CL	GM	AM	CP	LV	TC	TS	RF	VA
A (34)	IV	254	N		1992 (11), 1993 (13), 1994 (5), 1995 (1), 1996 (3), 2003 (1)	0	0	8.8	0	0	20.1	32.3	5.9	91.2	0	100
B (49)	III	241	N		1992 (8), 1993 (11), 1994 (10), 1995 (17), 1996 (3)	0	0	40.8	0	0	4.1	4.1	0	2.0	18.4	100
C (69)	III	239	N		1994 (1), 1995 (14), 1996 (34), 2003 (20)	0	0	5.8	2.9	0	1.4	1.4	2.9	1.4	95.7	100
D (6)	IV	59	Y		1994 (1), 1995 (2), 1996 (1), 2003 (2)	0	0	0	16.7	33.3	66.7	100	33.3	100	100	100
E (2)	IV	254	N		1992 (1), 1995 (1)	0	0	50	0	0	50	50	0	100	0	100
F (3)	IV	30	Y		1992 (2), 1993 (1)	0	0	0	33.3	66.7	100	100	33.3	100	100	100
K (11)	III	239	N		2003 (11)	0	0	9.1	0	0	0	0	0	0	100	100
L (5)	III	239	N		2003 (5)	0	0	0	0	0	0	0	0	0	100	100
M (8)	II	5	N		2003 (8)	0	0	0	0	0	0	0	100	100	25	100
Other‡ (3)																
	I	5		N	2003 (1)	0	0	0	0	0	0	0	100	100	0	100
	IV	59		Y	2003 (1)	0	0	0	0	100	100	100	100	100	100	100
	IV	59		Y	2003 (1)	0	0	0	0	0	100	100	0	100	100	100

*P, pulsotype; SCC, staphylococcal cassette chromosome; MLST, multilocus sequence typing; PVL, Panton-Valentine leukocidin; N, no; Y, yes. †OX, oxacillin; EM, erythromycin; CL, clinidamycin; GM, gentamicin; AM, amikacin; CP, ciprofloxacin; LV, levofloxacin; TC, tetracycline; TS, trimethoprim-sulfamethoxazole; RF, rifampin; VA, vancomycin. ‡Including 3 pulsotypes each containing only 1 isolate.

Results of MLST and typing of SCC*mec* elements of the 3 isolates with 3 minor pulsotypes obtained in 2003 are shown in Table 2. The correlation between SCC*mec* element typing and MLST in this study corresponds to findings of previous reports (*6*,*12*–*14*). Enright et al. identified ST254-IV MRSA isolates in Germany and the United Kingdom (*12*), and Chongtrakool et al. identified ST239-III and ST241-III MRSA isolates in several Asian countries (*14*).

## Conclusions

We demonstrate that the predominant MRSA clone at NTUH in early 1990s had the type IV SCC*mec* element. However, the predominant MRSA clones at NTUH from 1994 to 2003 had the type III SCC*mec* element. These findings differ from those of Wisplinghoff et al., who reported that that the SCC*mec* element in predominant MRSA clones at their institute changed from type III in 1984 to 1988 to type I in 1989 to 1998 (*6*). Differences between our findings and those of Wisplinghoff et al. might be caused by differences in location and epidemiologic characteristics.

MRSA isolates of pulsotypes B and C are more resistant than isolates of pulsotype A to certain antimicrobial drugs, especially fluoroquinolones and trimethoprim-sulfamethoxazole; MRSA isolates with pulsotype C are more resistant to clindamycin but less resistant to rifampin than those with pulsotype B (Table 2). From 1993 through 2000, annual use of fluoroquinolones increased ≈3× at NTUH; however, use of trimethoprim-sulfamethoxazole, clindamycin, and rifampin did not change (*15*). Therefore, the shift of predominant MRSA clones, which also led to the shift in types of SCC*mec* elements at NTUH, might be caused by selective pressure from antimicrobial drugs, especially fluoroquinolones.

The MRSA clone (pulsotype A) that predominated in 1992 and 1993 at NTUH has the type IV SCC*mec* element. Although the first study of MRSA with the type IV SCC*mec* element reported that this element was found in community-acquired MRSA (CA-MRSA) (*5*), some studies have reported MRSA isolates with this element in a hospital environment (*12*). However, to our knowledge, these reports did not demonstrate that MRSA isolates with the type IV SCC*mec* element became predominant among all MRSA isolates in an institution, especially before the mid-1990s.

Furthermore, 4 ST59 MRSA isolates obtained in 1994 and 1996 and 3 ST30 MRSA isolates obtained in 1992 and 1993 have the type IV SCC*mec* element and PVL gene. Recently, ST59 MRSA isolates were found to cause CA-MRSA infection in Taiwan (*13*). Among these ST59 CA-MRSA isolates, some had the type IV SCC*mec* element, and others had the type V SCC*mec* element (*13*). Although the type IV SCC*mec* element could be transferred to CA-MRSA clones with other genetic backgrounds, our finding supports the possibility that ST59 MRSA isolates with the SCC*mec* element type IV in Taiwan may originate from hospital strains but transfer into CA-MRSA strains.

Chongtrakool et al. recently reported the results of SCC*mec* typing of 615 MRSA isolates obtained in 1998 and 1999 from 11 Asian countries (*14*). The ST239-III, ST241-III, ST254-II, and ST5-II MRSA isolates were prevalent in many Asian countries. However, the ST254-IV, ST30-IV, and ST59-IV MRSA isolates from our study were not found in other Asian countries. In addition, ST254-IV MRSA isolates have been found in Germany and the United Kingdom (*12*). Whether ST254-IV MRSA isolates in our study were transmitted from those in Germany or the United Kingdom by international travel requires further study.

The first MRSA isolate with the type IV SCC*mec* element in our hospital appeared as early as 1992. The SCC*mec* element carried by predominant MRSA clones changed from type IV to type III SCC*mec* element during the period 1992–2003 at NTUH. Because the major MRSA clones isolated in 1994–2003 are more resistant to antimicrobial drugs, especially fluoroquinolones and trimethoprim-sulfamethoxazole, than those obtained in 1992 and 1993, this shift may be caused by selective pressure from indiscriminate use of antimicrobial drugs.
